# Individual- and Community-Level Risk Factors of Cancer-Related Financial Hardship Among Cancer Survivors

**DOI:** 10.1001/jamanetworkopen.2024.29286

**Published:** 2024-08-20

**Authors:** Apoorv Dhir, Kristian Donald Stensland, Lindsey Allison Herrel, Rishi Robert Sekar

**Affiliations:** 1 Department of Urology, University of Michigan, Ann Arbor; 2 Institute for Healthcare Policy and Innovation, University of Michigan, Ann Arbor; 3 National Clinician Scholars Program, University of Michigan, Ann Arbor

## Abstract

This cross-sectional study estimates the prevalence of financial hardship among cancer survivors and investigates its associations with individual- and community-level characteristics.

## Introduction

Cancer survivors face substantial economic hardships during and after oncologic care, compounding the physical and psychosocial implications of a cancer diagnosis and compromising treatment adherence, quality of life, and survival.^1,2^ Financial hardship has been the focus of policy initiatives addressing cost-sharing and insurance coverage to alleviate direct costs to patients.^3^ However, the role of community factors and structural barriers in the experience of financial hardship remains unknown. We hypothesized that adverse community-level social determinants of health may exacerbate financial hardship for socioeconomically disadvantaged populations.^4^ For these reasons, we estimated the prevalence of financial hardship among cancer survivors across cancer types and investigated associations with individual- and community-level characteristics.

## Methods

In this cross-sectional study, we analyzed the 2021 Health Information National Trends Survey–Surveillance, Epidemiology, and End Results (HINTS-SEER) (eAppendix in Supplement 1), which is sampled from 3 SEER registries.^5^ We included respondents of all ages, cancer types, and disease stages in the HINTS-SEER cohort. The primary outcome was patient-reported experience of cancer-related financial hardship (categorized as not at all, a little, some, or a lot). The primary exposure was the Social Vulnerability Index (SVI).^6^ Covariates included age, sex, education level, income, insurance status, and SEER cancer stage; individual-level race and ethnicity were omitted because community-level race and ethnicity are factored into the SVI and considered a social construct. Survey-weighted estimates of financial hardship across cancer types and for the entire cohort were calculated. Multivariable ordinal logistic regression for financial hardship was performed adjusting for all independent variables. Odds ratios were calculated for covariates, and average marginal effects were calculated for SVI. Analyses were adjusted for sample weights and complex survey design. Statistical analyses were performed with Stata, version 18 (StataCorp LLC); statistical significance was defined as 2-tailed *P* < .05.

Because HINTS-SEER data are publicly available, deidentified, and approved by local SEER registries and Westat Institutional Review Boards, this study was exempt from assessment and informed consent by the University of Michigan Institutional Review Board. We followed the STROBE reporting guideline.

## Results

**Figure 1.**
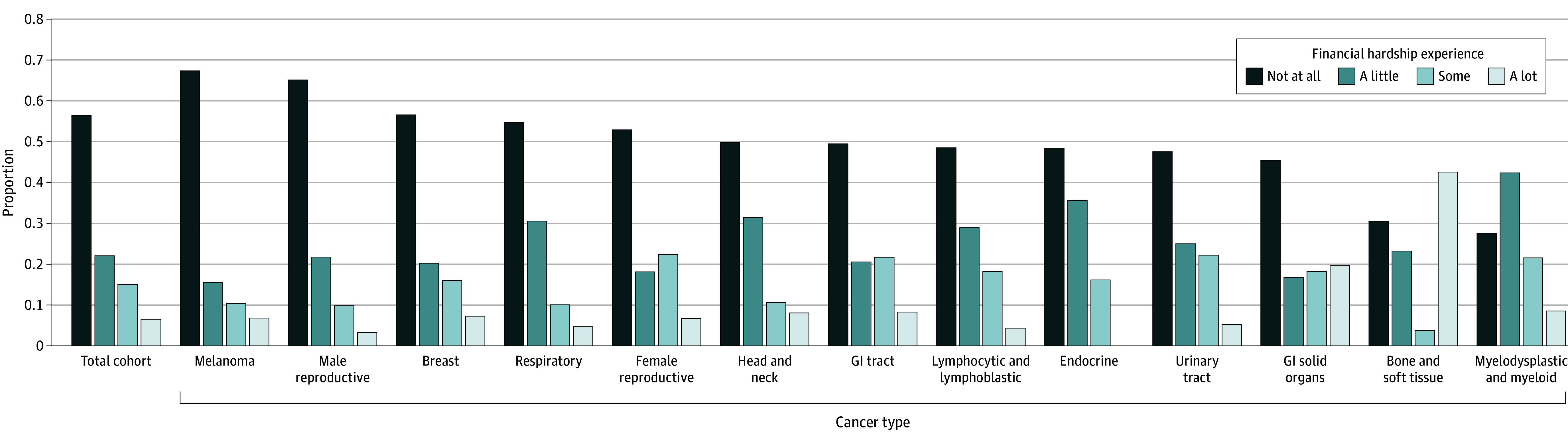
Survey-Weighted Proportions of Cancer Survivors Reporting Cancer-Related Financial Hardship by Cancer Type Results are shown for cancer types with 10 or more survey respondents per cancer type; therefore, the requisite data for unknown primary cancer, myeloma and plasma cell disorders, cancers of the central nervous system and meninges, and eye cancers are not shown.

**Figure 2.**
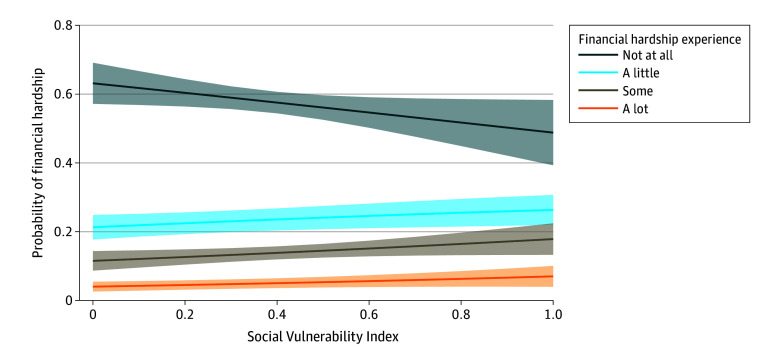
Adjusted Probability of Cancer-Related Financial Hardship by Social Vulnerability Index Increasing Social Vulnerability Index scores were independently associated with a lower probability of reporting no financial hardship and higher probability of reporting a little, some, or a lot of hardship, adjusting for age, sex, income, education level, insurance type, and cancer stage. Adjusted probabilities of reporting financial hardship by Social Vulnerability Index score were calculated using estimates from the multivariable ordinal logistic regression model. Lines indicate average marginal effects; shaded areas represent 95% CIs.

We identified 1212 respondents representing more than 400 000 cancer survivors on weighted analysis. The median (IQR) age was 72 years (65-79 years) and the median (IQR) SVI score was 0.41 (0.27-0.68); 59.1% had completed college, 56.5% had an annual household income greater than $75 000, 70.5% had private or employer-based health insurance, and 67.8% had localized disease. When asked about experience of financial hardship, 56.4%, 22.1%, 15.0%, and 6.5% responded not at all, a little, some, and a lot, respectively, with variation by cancer type (Figure 1). Increasing SVI score (adjusted odds ratio [aOR], 1.80 [95% CI, 1.00-3.21]), younger age (50-59 years; aOR, 3.66 [95% CI, 2.37-5.67]), lower income ($35 000-$74 999; aOR, 3.55 [95% CI, 1.94-6.48]), advanced SEER cancer stage (nonlocal; aOR, 1.92 [95% CI, 1.47-2.51]), and federal health insurance (Medicare; aOR, 1.43 [95% CI, 1.02-2.00]) were associated with higher odds of increased financial hardship. An increase in SVI of 0.01 conferred a lower probability of reporting no financial hardship and higher probability of reporting a little, some, or a lot of financial hardship (average marginal effects: −12.37 [95% CI, −24.58 to −0.15], 3.49 [95% CI, 0.04-7.02], 5.35 [95% CI, 0.12-10.57], and 3.53 [95% CI, −0.13-7.20], respectively) (Figure 2).

## Discussion

In this cross-sectional study, approximately 50% of cancer survivors experienced cancer-related financial hardship, with a disproportionate number of survivors who were younger, were Medicare or Medicaid beneficiaries, were socioeconomically disadvantaged, and had advanced disease. Social vulnerability was independently associated with increasing levels of financial hardship, emphasizing the central role one’s community may play in cancer survivorship and the compounding financial detriments of residing in a socially vulnerable community.

Study limitations include a study design that precluded our ability to establish causality, selection bias and reduced generalizability due to sampling methodology, recall bias due to patient-reported variables, and unobserved confounding. Although policy efforts to curb the costs of cancer care are essential, acknowledging the role of community and structural barriers and identifying socially vulnerable populations for targeted interventions represent a potential strategy toward mitigating financial hardship and delivering equitable cancer care.

## Notes

Supplement 1.
eAppendix.

Supplement 2.
Data Sharing Statement
