# Supplementary material for: Individual- and Community-Level Risk Factors of Cancer-Related Financial Hardship Among Cancer Survivors
Source: JAMA Netw Open. 2024 Aug 20;7(8):e2429286. doi: 10.1001/jamanetworkopen.2024.29286 (PMC11337072; doi:10.1001/jamanetworkopen.2024.29286)
Supplement: Supplement 1. — eAppendix. [file jamanetwopen-e2429286-s001.pdf]

Supplemental Online Content

Dhir A, Stensland KD, Herrel LA, Sekar RR. Individual- and community-level risk factors of cancer-related financial hardship among cancer survivors. *JAMA Netw Open*. 2024;7(8):e2429286. doi:10.1001/jamanetworkopen.2024.29286

eAppendix

This supplemental material has been provided by the authors to give readers additional information about their work.

© 2024 Dhir A et al. *JAMA Network Open.*

eAppendix

The Health Information National Trends Survey – Surveillance, Epidemiology, End Results (HINTS-SEER) is a pilot study conducted by the National Cancer Institute to oversample cancer survivors using the HINTS survey instrument (HINTS 5 Cycle 4, 2020). Cancer survivors were sampled from three SEER registries (Iowa, New Mexico, and the Greater Bay Area). Cancer survivors that met the following criteria were included in the sampling frame: 1) vital status of alive, 2) older than 18 years of age, 3) last contact no earlier than January 1, 2016, and 4) date of cancer diagnosis prior to 2018. Survivors with non-melanoma skin cancers were excluded. Stratified sampling was performed for each SEER registry by year since diagnosis and race/ethnicity. A total of 9,826 cancer survivors were sampled, of which 1,806 (18.4%) consented to participate, and 1,234 (68.3%) completed the survey, for an overall response rate of 12.6%. HINTS-SEER received approval from the Westat Institutional Review Board. Each participating SEER registry obtained independent Institutional Review Board approval.

This study focused on survey question E7 ("Looking back, since the time you were first diagnosed with cancer, how much, if at all, has cancer and its treatment hurt your financial situation"). Responses included "Not at all", "A little", "Some", and "A lot". Of the 1,234 respondents, 22 (1.78%) had a missing response for the survey question of interest and were therefore excluded from the analysis.

© 2024 Dhir A et al. *JAMA Network Open.*
