# Supplementary material for: Individual- and Community-Level Risk Factors of Cancer-Related Financial Hardship Among Cancer Survivors
Source: JAMA Netw Open. 2024 Aug 20;7(8):e2429286. doi: 10.1001/jamanetworkopen.2024.29286 (PMC11337072; doi:10.1001/jamanetworkopen.2024.29286)
Supplement: Supplement 2. — Data Sharing Statement [file jamanetwopen-e2429286-s002.pdf]

Data Sharing Statement

Dhir. Individual- and Community-Level Risk Factors of Cancer-Related Financial Hardship Among Cancer Survivors. *JAMA Netw Open*. Published August 20, 2024. doi:10.1001/jamanetworkopen.2024.29286

Data
Data available: No

Additional Information
Explanation for why data not available: Data is publicly available at https://hints.cancer.gov/.
